# Methyl *N*′-[(*E*)-2-methoxy­benzyl­idene]­hydrazinecarboxyl­ate

**DOI:** 10.1107/S1600536809014172

**Published:** 2009-04-22

**Authors:** Lu-Ping Lv, Wen-Bo Yu, Zhong-Hao Lu, Wei-Wei Li, Xian-Chao Hu

**Affiliations:** a Department of Chemical Engineering, Hangzhou Vocational and Technical College, Hangzhou 310018, People’s Republic of China; b Air Liquide (Hangzhou) Co. Ltd, Hangzhou 311112, People’s Republic of China; c Research Center of Analysis and Measurement, Zhejiang University of Technology, Hangzhou 310014, People’s Republic of China

## Abstract

The title compound, C_10_H_12_N_2_O_3_, crystallizes with two independent mol­ecules in the asymmetric unit. The side chains in the two independent mol­ecules have slightly different orientations, with the C=N—N—C torsion angle being 169.19 (14)° in one of the mol­ecules and −179.86 (14)° in the other. Each independent mol­ecule adopts a *trans* configuration with respect to the C=N bond. In the crystal structure, mol­ecules are linked into chains running along [001] by N—H⋯O, N—H⋯N and C—H⋯O hydrogen bonds. In addition, an inter­molecular C—H⋯π inter­action is observed.

## Related literature

For applications of benzaldehydehydrazone derviatives, see: Parashar *et al.* (1988 ▶); Hadjoudis *et al.* (1987 ▶); Borg *et al.* (1999 ▶). For metal complexes of Schiff base ligands, see: Kahwa *et al.* (1986 ▶); Santos *et al.* (2001 ▶). For a related structure, see: Shang *et al.* (2007 ▶).
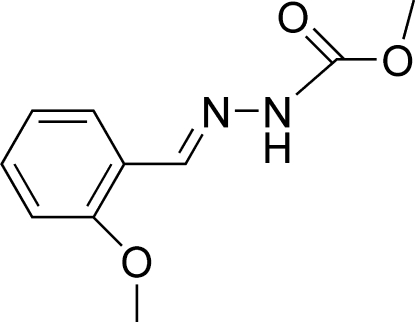


## Experimental

### 

#### Crystal data

C_10_H_12_N_2_O_3_
*M* *_r_* = 208.22
Monoclinic, 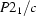

*a* = 17.221 (5) Å
*b* = 7.442 (2) Å
*c* = 16.611 (6) Å
β = 95.423 (12)°
*V* = 2119.4 (12) Å^3^
*Z* = 8
Mo *K*α radiation
μ = 0.10 mm^−1^
*T* = 223 K
0.24 × 0.21 × 0.19 mm

#### Data collection

Bruker SMART CCD area-detector diffractometer
Absorption correction: multi-scan (*SADABS*; Bruker, 2002 ▶) *T*_min_ = 0.977, *T*_max_ = 0.989
11064 measured reflections
3723 independent reflections
2834 reflections with *I* > 2σ(*I*)
*R*_int_ = 0.026

#### Refinement

*R*[*F* ^2^ > 2σ(*F* ^2^)] = 0.041
*wR*(*F* ^2^) = 0.120
*S* = 1.08
3723 reflections
271 parameters
H-atom parameters constrained
Δρ_max_ = 0.21 e Å^−3^
Δρ_min_ = −0.18 e Å^−3^

### 

Data collection: *SMART* (Bruker, 2002 ▶); cell refinement: *SAINT* (Bruker, 2002 ▶); data reduction: *SAINT*; program(s) used to solve structure: *SHELXS97* (Sheldrick, 2008 ▶); program(s) used to refine structure: *SHELXL97* (Sheldrick, 2008 ▶); molecular graphics: *SHELXTL* (Sheldrick, 2008 ▶); software used to prepare material for publication: *SHELXTL*.

## Supplementary Material

Crystal structure: contains datablocks I, global. DOI: 10.1107/S1600536809014172/ci2777sup1.cif

Structure factors: contains datablocks I. DOI: 10.1107/S1600536809014172/ci2777Isup2.hkl

Additional supplementary materials:  crystallographic information; 3D view; checkCIF report

## Figures and Tables

**Table 1:** Hydrogen-bond geometry (Å, °) *Cg*1 is the centroid of the C12–C17 ring.

*D*—H⋯*A*	*D*—H	H⋯*A*	*D*⋯*A*	*D*—H⋯*A*
N2—H2N⋯O5	0.86	2.08	2.938 (2)	173
N4—H4N⋯N1^i^	0.86	2.42	3.279 (2)	177
C1—H1*A*⋯O2^i^	0.96	2.52	3.472 (2)	170
C11—H11*B*⋯*Cg*1^ii^	0.96	2.87	3.826 (3)	175

Symmetry codes: (i) 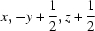; (ii) 
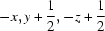
.

**Fig. 1.**
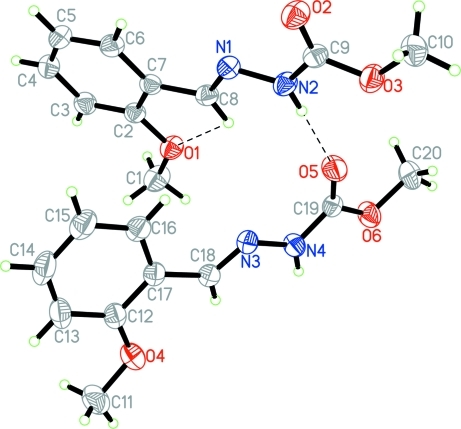
The asymmetric unit of the title compound. Displacement ellipsoids are drawn at the 30% probability level. Dashed lines indicate hydrogen bonds.

**Fig. 2.**
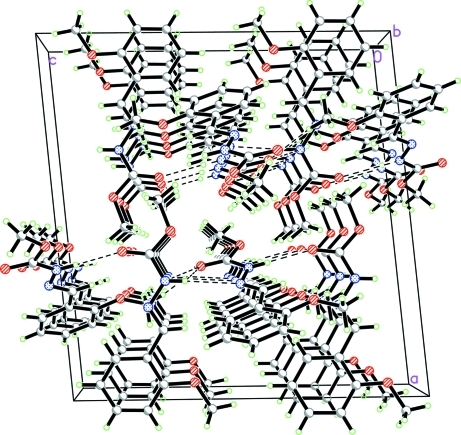
Crystal packing of the title compound. Hydrogen bonds are shown as dashed lines.

## supplementary crystallographic information

### Comment

Benzaldehydehydrazone derivatives have attracted much attention due to their pharmacological activity (Parashar *et al.*, 1988) and their photochromic properties (Hadjoudis *et al.*, 1987). They are important intermediates of 1,3,4-oxadiazoles, which have been reported to be versatile compounds with many interesting properties (Borg *et al.*, 1999). Metal complexes based on Schiff bases have received considerable attention because they can be utilized as model compounds of active centres in various proteins and enzymes (Kahwa *et al.*, 1986; Santos *et al.*, 2001). We report here the crystal structure of the title compound (Fig. 1).

The title compound contains two independent, but almost identical molecules in the asymmetric unit. Each independent molecule adopts a *trans* configuration with respect to the C═N bond. The N1/N2/O2/O3/C8/C9 and N3/N4/O5/O6/C18/C19 planes form dihedral angles of 3.20 (6)° and 11.61 (5)°, respectively, with the C2—C7 and C12—C17 planes. The dihedral angle between the two independent benzene rings is 49.19 (7)°. The bond lengths and angles are comparable to those observed for methyl*N*'-[(*E*)-4-methoxybenzylidene]hydrazinecarboxylate (Shang *et al.*, 2007).

In the crystal structure, the molecules are linked into chains running along the [001] by N—H···O, N—H···N and C—H···O hydrogen bonds. In addition, an intermolecular C—H···π interaction is observed (Table 1).

### Experimental

2-Methoxybenzaldehyde (1.36 g, 0.01 mol) and methyl hydrazinecarboxylate (0.90 g, 0.01 mol) were dissolved in stirred methanol (25 ml) and left for 2.5 h at room temperature. The resulting solid was filtered off and recrystallized from ethanol to give the title compound in 95% yield. Single crystals suitable for X-ray analysis were obtained by slow evaporation of an ethanol solution at room temperature (m.p. 428–430 K).

### Refinement

H atoms were positioned geometrically (N-H = 0.86 Å and C-H = 0.93 or 0.96 Å) and refined using a riding model, with *U*_iso_(H) = 1.2*U*_eq_(C,N) and 1.5*U*_eq_(C_methyl_). In the absence of significant anomalous scattering effects, Friedel pairs were averaged.

### Crystal data

**Table:**

C_10_H_12_N_2_O_3_	*F*(000) = 880
*M**_r_* = 208.22	*D*_x_ = 1.305 Mg m^−^^3^
Monoclinic, *P*2_1_/*c*	Mo *K*α radiation, λ = 0.71073 Å
Hall symbol: -P 2ybc	Cell parameters from 3723 reflections
*a* = 17.221 (5) Å	θ = 1.2–25.0°
*b* = 7.442 (2) Å	µ = 0.10 mm^−^^1^
*c* = 16.611 (6) Å	*T* = 223 K
β = 95.423 (12)°	Block, colourless
*V* = 2119.4 (12) Å^3^	0.24 × 0.21 × 0.19 mm
*Z* = 8	

### Data collection

**Table:**

Bruker SMART CCD area-detector diffractometer	3723 independent reflections
Radiation source: fine-focus sealed tube	2834 reflections with *I* > 2σ(*I*)
graphite	*R*_int_ = 0.026
φ and ω scans	θ_max_ = 25.0°, θ_min_ = 1.2°
Absorption correction: multi-scan (*SADABS*; Bruker, 2002)	*h* = −20→20
*T*_min_ = 0.977, *T*_max_ = 0.989	*k* = −8→8
11064 measured reflections	*l* = −19→18

### Refinement

**Table:**

Refinement on *F*^2^	Primary atom site location: structure-invariant direct methods
Least-squares matrix: full	Secondary atom site location: difference Fourier map
*R*[*F*^2^ > 2σ(*F*^2^)] = 0.041	Hydrogen site location: inferred from neighbouring sites
*wR*(*F*^2^) = 0.120	H-atom parameters constrained
*S* = 1.08	*w* = 1/[σ^2^(*F*_o_^2^) + (0.0639*P*)^2^ + 0.1492*P*] where *P* = (*F*_o_^2^ + 2*F*_c_^2^)/3
3723 reflections	(Δ/σ)_max_ = 0.001
271 parameters	Δρ_max_ = 0.21 e Å^−^^3^
0 restraints	Δρ_min_ = −0.18 e Å^−^^3^

### Special details

**Table:**

Geometry. All e.s.d.'s (except the e.s.d. in the dihedral angle between two l.s. planes) are estimated using the full covariance matrix. The cell e.s.d.'s are taken into account individually in the estimation of e.s.d.'s in distances, angles and torsion angles; correlations between e.s.d.'s in cell parameters are only used when they are defined by crystal symmetry. An approximate (isotropic) treatment of cell e.s.d.'s is used for estimating e.s.d.'s involving l.s. planes.
Refinement. Refinement of *F*^2^ against ALL reflections. The weighted *R*-factor *wR* and goodness of fit *S* are based on *F*^2^, conventional *R*-factors *R* are based on *F*, with *F* set to zero for negative *F*^2^. The threshold expression of *F*^2^ > σ(*F*^2^) is used only for calculating *R*-factors(gt) *etc*. and is not relevant to the choice of reflections for refinement. *R*-factors based on *F*^2^ are statistically about twice as large as those based on *F*, and *R*- factors based on ALL data will be even larger.

### Fractional atomic coordinates and isotropic or equivalent isotropic displacement parameters (Å^2^)

**Table:**

	*x*	*y*	*z*	*U*_iso_*/*U*_eq_	
O1	0.27964 (7)	−0.12236 (16)	0.66716 (7)	0.0579 (3)	
O3	0.41712 (7)	0.63135 (17)	0.50573 (7)	0.0644 (4)	
O2	0.36311 (7)	0.49059 (17)	0.39394 (7)	0.0649 (4)	
O6	0.45355 (6)	0.3646 (2)	0.82317 (7)	0.0676 (4)	
O4	0.06692 (7)	0.15183 (19)	0.84498 (9)	0.0716 (4)	
N3	0.26187 (7)	0.29235 (18)	0.74487 (8)	0.0478 (3)	
O5	0.40316 (7)	0.3884 (2)	0.69432 (8)	0.0733 (4)	
N1	0.31881 (7)	0.21928 (19)	0.49137 (8)	0.0483 (3)	
N2	0.35854 (8)	0.37207 (19)	0.51936 (8)	0.0522 (4)	
H2N	0.3707	0.3869	0.5703	0.063*	
N4	0.32825 (7)	0.3208 (2)	0.79597 (8)	0.0525 (4)	
H4N	0.3270	0.3140	0.8475	0.063*	
C7	0.26100 (8)	−0.0607 (2)	0.52867 (9)	0.0460 (4)	
C17	0.13105 (9)	0.1853 (2)	0.72717 (11)	0.0506 (4)	
C18	0.20343 (9)	0.2284 (2)	0.77764 (10)	0.0497 (4)	
H18	0.2067	0.2093	0.8332	0.060*	
C8	0.30274 (8)	0.1085 (2)	0.54641 (10)	0.0479 (4)	
H8	0.3183	0.1366	0.6001	0.057*	
C9	0.37808 (9)	0.4974 (2)	0.46625 (10)	0.0492 (4)	
C16	0.12959 (10)	0.1783 (3)	0.64363 (12)	0.0616 (5)	
H16	0.1746	0.2063	0.6194	0.074*	
C4	0.18349 (10)	−0.3883 (3)	0.50054 (12)	0.0635 (5)	
H4	0.1581	−0.4976	0.4910	0.076*	
C3	0.21174 (10)	−0.3421 (2)	0.57816 (11)	0.0569 (5)	
H3	0.2052	−0.4199	0.6208	0.068*	
C5	0.19269 (10)	−0.2740 (3)	0.43723 (11)	0.0630 (5)	
H5	0.1730	−0.3054	0.3851	0.076*	
C1	0.26395 (13)	−0.2246 (3)	0.73518 (11)	0.0735 (6)	
H1A	0.2882	−0.1691	0.7833	0.110*	
H1B	0.2843	−0.3438	0.7304	0.110*	
H1C	0.2086	−0.2306	0.7382	0.110*	
C2	0.25007 (8)	−0.1791 (2)	0.59263 (10)	0.0470 (4)	
C20	0.53167 (10)	0.3890 (3)	0.80043 (15)	0.0834 (7)	
H20A	0.5678	0.3901	0.8481	0.125*	
H20B	0.5348	0.5010	0.7722	0.125*	
H20C	0.5444	0.2922	0.7658	0.125*	
C10	0.44366 (12)	0.7734 (3)	0.45624 (14)	0.0771 (6)	
H10A	0.4707	0.8621	0.4901	0.116*	
H10B	0.3997	0.8279	0.4258	0.116*	
H10C	0.4783	0.7247	0.4198	0.116*	
C6	0.23130 (10)	−0.1116 (3)	0.45094 (11)	0.0578 (5)	
H6	0.2375	−0.0354	0.4076	0.069*	
C14	−0.00378 (13)	0.0922 (3)	0.63078 (16)	0.0853 (7)	
H14	−0.0489	0.0620	0.5984	0.102*	
C19	0.39555 (9)	0.3595 (2)	0.76374 (10)	0.0477 (4)	
C13	−0.00495 (11)	0.0976 (3)	0.71332 (16)	0.0752 (6)	
H13	−0.0507	0.0708	0.7365	0.090*	
C12	0.06241 (9)	0.1433 (2)	0.76246 (12)	0.0582 (5)	
C15	0.06312 (12)	0.1308 (3)	0.59552 (14)	0.0783 (6)	
H15	0.0636	0.1250	0.5396	0.094*	
C11	−0.00264 (13)	0.1165 (3)	0.88362 (16)	0.0942 (8)	
H11A	0.0085	0.1268	0.9412	0.141*	
H11B	−0.0208	−0.0028	0.8703	0.141*	
H11C	−0.0422	0.2019	0.8652	0.141*	

### Atomic displacement parameters (Å^2^)

**Table:**

	*U*^11^	*U*^22^	*U*^33^	*U*^12^	*U*^13^	*U*^23^
O1	0.0726 (7)	0.0557 (8)	0.0444 (7)	−0.0051 (6)	0.0008 (5)	0.0033 (5)
O3	0.0752 (8)	0.0550 (8)	0.0625 (8)	−0.0139 (6)	0.0050 (6)	−0.0052 (6)
O2	0.0852 (8)	0.0594 (9)	0.0491 (8)	−0.0064 (6)	0.0008 (6)	0.0032 (6)
O6	0.0470 (6)	0.0963 (11)	0.0579 (8)	−0.0085 (6)	−0.0042 (5)	0.0026 (7)
O4	0.0582 (7)	0.0764 (10)	0.0818 (10)	−0.0074 (6)	0.0150 (6)	0.0087 (7)
N3	0.0471 (7)	0.0452 (8)	0.0496 (8)	0.0012 (6)	−0.0030 (6)	0.0025 (6)
O5	0.0682 (8)	0.1061 (12)	0.0457 (8)	−0.0207 (7)	0.0058 (6)	0.0028 (7)
N1	0.0514 (7)	0.0468 (9)	0.0464 (8)	0.0006 (6)	0.0027 (6)	−0.0005 (7)
N2	0.0624 (8)	0.0539 (10)	0.0392 (7)	−0.0041 (7)	−0.0002 (6)	−0.0016 (6)
N4	0.0462 (7)	0.0693 (10)	0.0408 (7)	−0.0045 (6)	−0.0018 (6)	0.0058 (7)
C7	0.0417 (8)	0.0506 (10)	0.0461 (9)	0.0046 (7)	0.0065 (6)	−0.0014 (8)
C17	0.0474 (8)	0.0382 (10)	0.0648 (11)	0.0069 (7)	−0.0018 (7)	−0.0028 (8)
C18	0.0489 (9)	0.0466 (10)	0.0528 (10)	0.0038 (7)	0.0009 (7)	0.0009 (8)
C8	0.0482 (8)	0.0540 (11)	0.0416 (9)	0.0036 (7)	0.0047 (7)	−0.0010 (8)
C9	0.0526 (9)	0.0483 (11)	0.0462 (10)	0.0050 (7)	0.0021 (7)	−0.0018 (8)
C16	0.0562 (10)	0.0587 (12)	0.0681 (12)	0.0111 (8)	−0.0036 (8)	−0.0107 (9)
C4	0.0579 (10)	0.0629 (13)	0.0704 (13)	−0.0092 (9)	0.0093 (9)	−0.0139 (10)
C3	0.0579 (10)	0.0535 (12)	0.0605 (11)	−0.0032 (8)	0.0110 (8)	0.0008 (9)
C5	0.0601 (10)	0.0758 (14)	0.0525 (11)	−0.0061 (9)	0.0012 (8)	−0.0149 (10)
C1	0.1031 (15)	0.0659 (14)	0.0504 (11)	−0.0079 (11)	0.0014 (10)	0.0094 (10)
C2	0.0439 (8)	0.0485 (10)	0.0488 (10)	0.0050 (7)	0.0058 (7)	−0.0025 (8)
C20	0.0483 (10)	0.0993 (18)	0.1017 (17)	−0.0118 (10)	0.0030 (10)	−0.0090 (14)
C10	0.0798 (13)	0.0608 (14)	0.0931 (16)	−0.0161 (10)	0.0202 (12)	−0.0003 (11)
C6	0.0569 (10)	0.0692 (13)	0.0477 (10)	−0.0006 (9)	0.0067 (8)	−0.0018 (9)
C14	0.0684 (13)	0.0708 (15)	0.1093 (19)	0.0035 (11)	−0.0308 (13)	−0.0254 (13)
C19	0.0511 (9)	0.0468 (10)	0.0441 (10)	−0.0031 (7)	−0.0004 (7)	−0.0015 (7)
C13	0.0499 (10)	0.0543 (13)	0.119 (2)	−0.0044 (9)	−0.0018 (11)	−0.0084 (12)
C12	0.0512 (9)	0.0415 (11)	0.0810 (14)	0.0022 (7)	0.0016 (9)	−0.0019 (9)
C15	0.0744 (13)	0.0752 (16)	0.0804 (14)	0.0126 (11)	−0.0187 (11)	−0.0219 (12)
C11	0.0803 (14)	0.0885 (18)	0.119 (2)	−0.0205 (12)	0.0373 (13)	0.0061 (15)

### Geometric parameters (Å, °)

**Table:**

O1—C2	1.3605 (19)	C16—H16	0.93
O1—C1	1.409 (2)	C4—C5	1.374 (3)
O3—C9	1.339 (2)	C4—C3	1.378 (3)
O3—C10	1.440 (2)	C4—H4	0.93
O2—C9	1.205 (2)	C3—C2	1.391 (2)
O6—C19	1.3370 (19)	C3—H3	0.93
O6—C20	1.443 (2)	C5—C6	1.388 (3)
O4—C12	1.367 (2)	C5—H5	0.93
O4—C11	1.436 (2)	C1—H1A	0.96
N3—C18	1.280 (2)	C1—H1B	0.96
N3—N4	1.3742 (17)	C1—H1C	0.96
O5—C19	1.1922 (19)	C20—H20A	0.96
N1—C8	1.281 (2)	C20—H20B	0.96
N1—N2	1.3846 (19)	C20—H20C	0.96
N2—C9	1.348 (2)	C10—H10A	0.96
N2—H2N	0.86	C10—H10B	0.96
N4—C19	1.353 (2)	C10—H10C	0.96
N4—H4N	0.86	C6—H6	0.93
C7—C6	1.395 (2)	C14—C15	1.371 (3)
C7—C2	1.406 (2)	C14—C13	1.374 (3)
C7—C8	1.466 (2)	C14—H14	0.93
C17—C16	1.386 (3)	C13—C12	1.396 (3)
C17—C12	1.403 (2)	C13—H13	0.93
C17—C18	1.471 (2)	C15—H15	0.93
C18—H18	0.93	C11—H11A	0.96
C8—H8	0.93	C11—H11B	0.96
C16—C15	1.379 (3)	C11—H11C	0.96
			
C2—O1—C1	118.57 (14)	O1—C1—H1C	109.5
C9—O3—C10	116.02 (15)	H1A—C1—H1C	109.5
C19—O6—C20	117.40 (15)	H1B—C1—H1C	109.5
C12—O4—C11	117.97 (16)	O1—C2—C3	123.94 (16)
C18—N3—N4	115.85 (13)	O1—C2—C7	115.33 (14)
C8—N1—N2	114.99 (13)	C3—C2—C7	120.73 (15)
C9—N2—N1	119.64 (13)	O6—C20—H20A	109.5
C9—N2—H2N	120.2	O6—C20—H20B	109.5
N1—N2—H2N	120.2	H20A—C20—H20B	109.5
C19—N4—N3	118.82 (13)	O6—C20—H20C	109.5
C19—N4—H4N	120.6	H20A—C20—H20C	109.5
N3—N4—H4N	120.6	H20B—C20—H20C	109.5
C6—C7—C2	117.76 (16)	O3—C10—H10A	109.5
C6—C7—C8	123.29 (16)	O3—C10—H10B	109.5
C2—C7—C8	118.96 (14)	H10A—C10—H10B	109.5
C16—C17—C12	118.26 (16)	O3—C10—H10C	109.5
C16—C17—C18	120.86 (16)	H10A—C10—H10C	109.5
C12—C17—C18	120.84 (16)	H10B—C10—H10C	109.5
N3—C18—C17	119.80 (15)	C5—C6—C7	121.06 (17)
N3—C18—H18	120.1	C5—C6—H6	119.5
C17—C18—H18	120.1	C7—C6—H6	119.5
N1—C8—C7	122.99 (15)	C15—C14—C13	120.82 (19)
N1—C8—H8	118.5	C15—C14—H14	119.6
C7—C8—H8	118.5	C13—C14—H14	119.6
O2—C9—O3	124.73 (16)	O5—C19—O6	124.49 (15)
O2—C9—N2	125.42 (16)	O5—C19—N4	126.70 (14)
O3—C9—N2	109.84 (14)	O6—C19—N4	108.79 (14)
C15—C16—C17	121.7 (2)	C14—C13—C12	120.1 (2)
C15—C16—H16	119.2	C14—C13—H13	120.0
C17—C16—H16	119.2	C12—C13—H13	120.0
C5—C4—C3	120.38 (18)	O4—C12—C13	124.25 (18)
C5—C4—H4	119.8	O4—C12—C17	116.00 (15)
C3—C4—H4	119.8	C13—C12—C17	119.75 (19)
C4—C3—C2	119.93 (17)	C14—C15—C16	119.4 (2)
C4—C3—H3	120.0	C14—C15—H15	120.3
C2—C3—H3	120.0	C16—C15—H15	120.3
C4—C5—C6	120.14 (17)	O4—C11—H11A	109.5
C4—C5—H5	119.9	O4—C11—H11B	109.5
C6—C5—H5	119.9	H11A—C11—H11B	109.5
O1—C1—H1A	109.5	O4—C11—H11C	109.5
O1—C1—H1B	109.5	H11A—C11—H11C	109.5
H1A—C1—H1B	109.5	H11B—C11—H11C	109.5

### Hydrogen-bond geometry (Å, °)

**Table:**

*D*—H···*A*	*D*—H	H···*A*	*D*···*A*	*D*—H···*A*
N2—H2N···O5	0.86	2.08	2.938 (2)	173
N4—H4N···N1^i^	0.86	2.42	3.279 (2)	177
C1—H1A···O2^i^	0.96	2.52	3.472 (2)	170
C11—H11B···Cg1^ii^	0.96	2.87	3.826 (3)	175

Symmetry codes: (i) *x*, −*y*+1/2, *z*+1/2; (ii) −*x*, *y*+1/2, −*z*+1/2.
